# Supplementary material for: The Reactive Sites of Methane Activation: A Comparison of IrC3+ with PtC3+
Source: Molecules. 2021 Oct 4;26(19):6028. doi: 10.3390/molecules26196028 (PMC8512126; doi:10.3390/molecules26196028)
Supplement: Supplementary file 1 [file molecules-26-06028-s001.zip › molecules-1399710-supplementary.pdf]

The Reactive Sites of Methane Activation: A Comparison of IrC_3^+ with PtC_3^+

Zizhuang Liu[1], Hechen Wu[1], Wei Li[2], Xiaonan Wu[1]*,

1. Department of Chemistry, Fudan University, Shanghai, 200433, China.

2. School of Mathematics and Physics, North China Electric Power University, Beinong Road 2, Huilongguan, Beijing 102206, China.

Authors to whom correspondence should be addressed: wuxiaonan@fudan.edu.cn

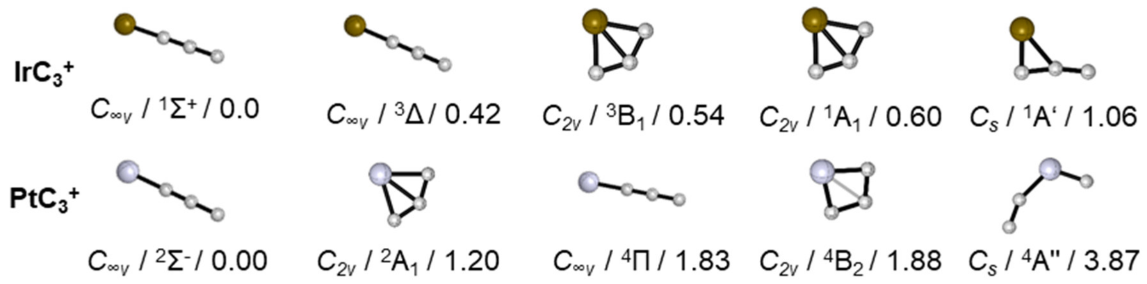

Figure S1. The optimized geometries of the $[IrC_3]^+$ and $[PtC_3]^+$ isomers at the BMK/def2-TZVP level. The relative energies relative to the global minimum structure (in eV), symmetry and electronic states are shown.

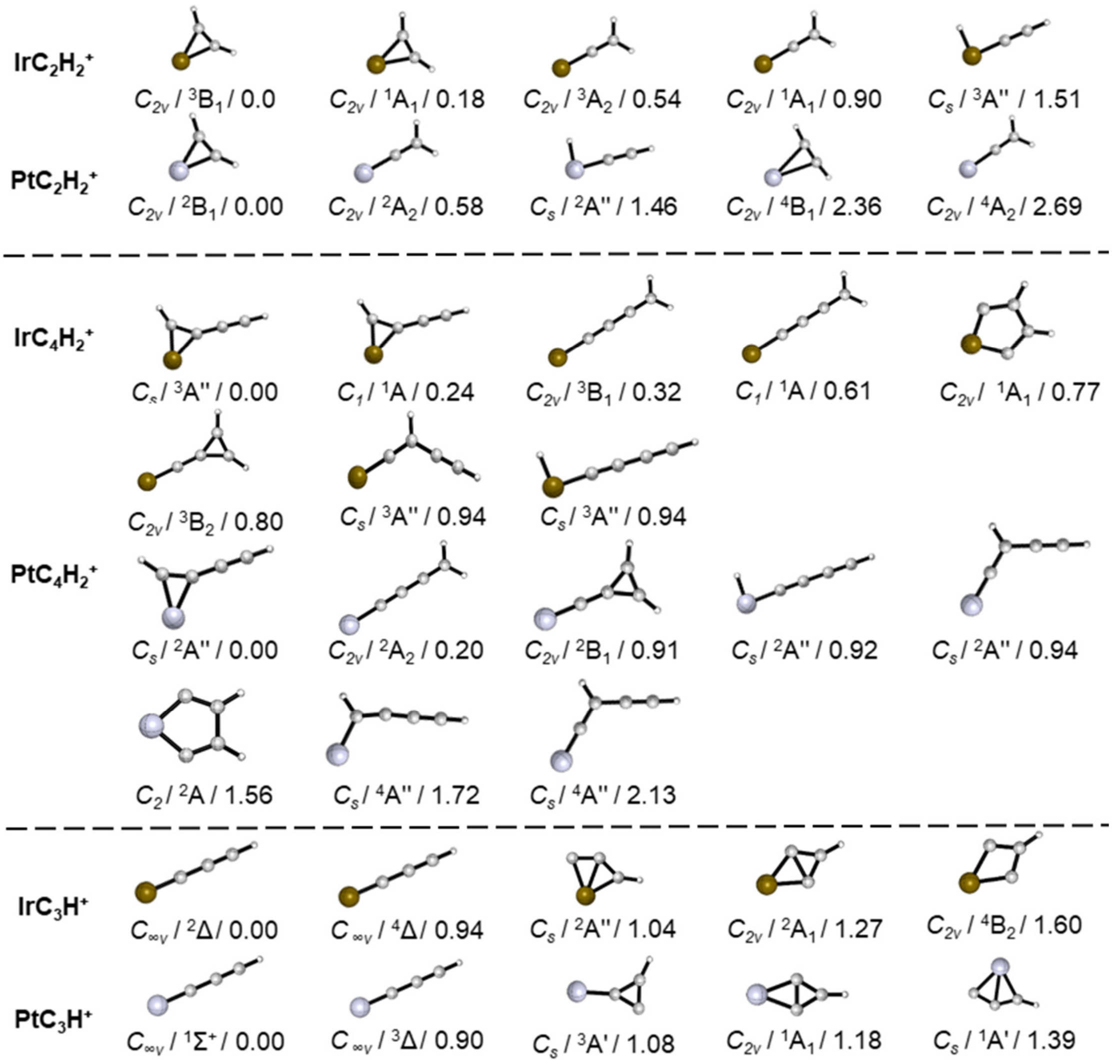

Figure S2. The optimized geometries of the $[IrC_2H_2]^+$/$[PtC_2H_2]^+$ (top), $[IrC_4H_2]^+$/$[PtC_4H_2]^+$ (middle) and $[IrC_3H]^+$/$[PtC_3H]^+$ (bottom) isomers at the BMK/def2-TZVP level. The relative energies relative to the global minimum structure (in eV), symmetry and electronic states are shown.

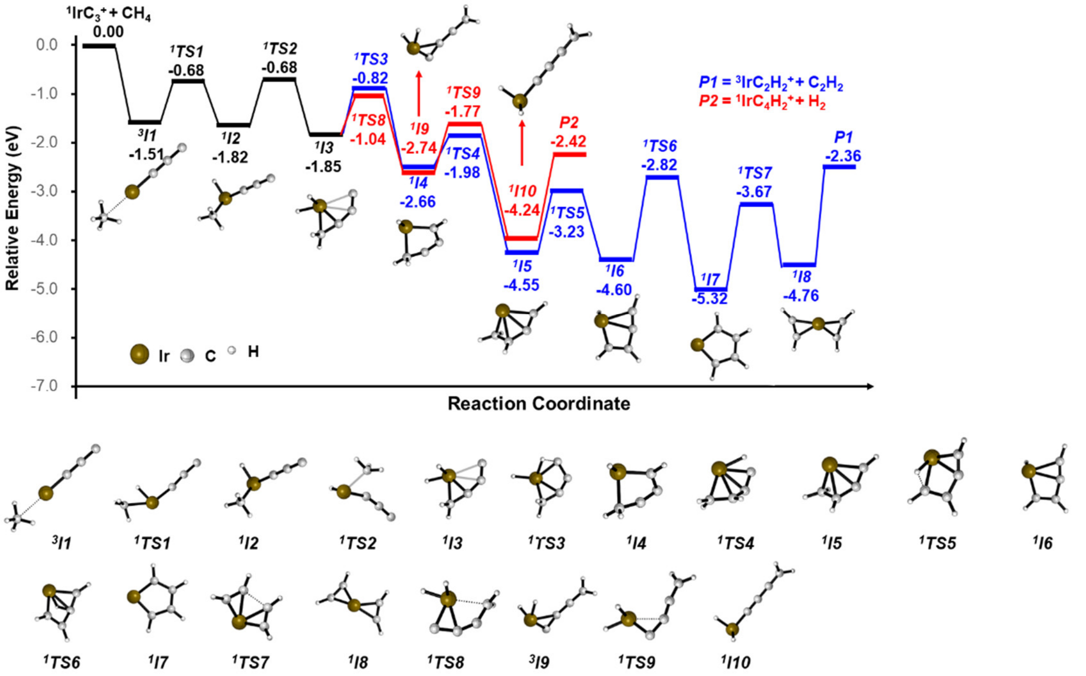

Figure S3. The detailed potential energy profiles of the reaction of IrC_3^+ and CH_4 in Figure 2. The energies are given in eV.

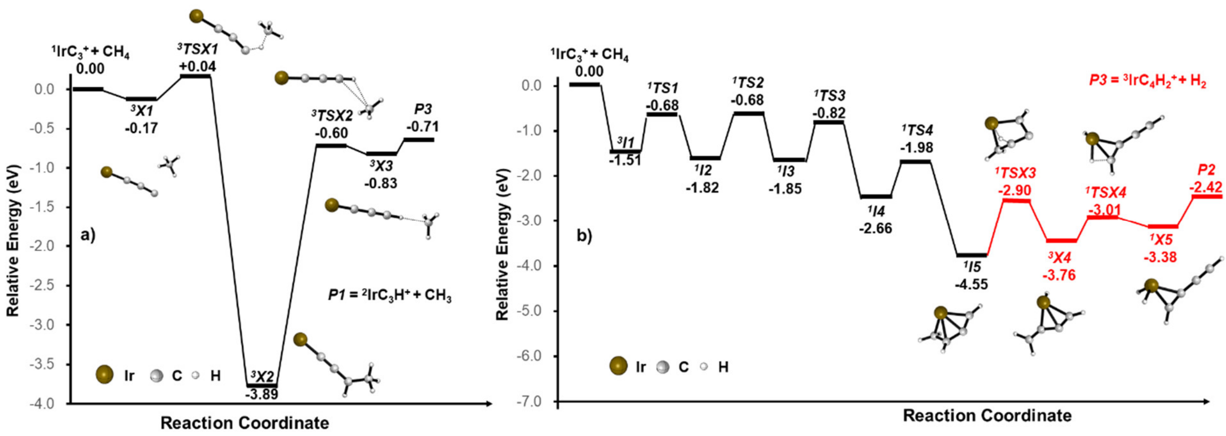

Figure S4. The other possible potential energy profiles of the reaction of IrC_3^+ and CH_4. The energies are given in eV.

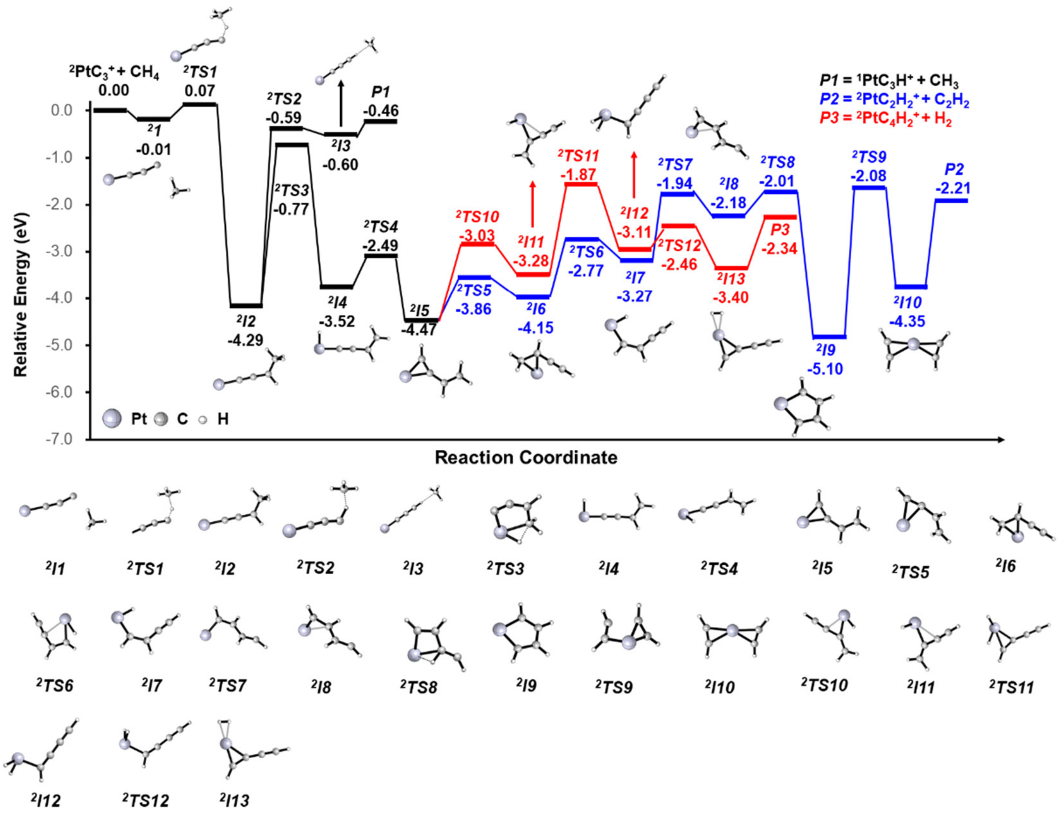

Figure S5. The detailed potential energy profiles of the reaction of PtC_3^+ and CH_4 in Figure 3. The energies are given in eV.

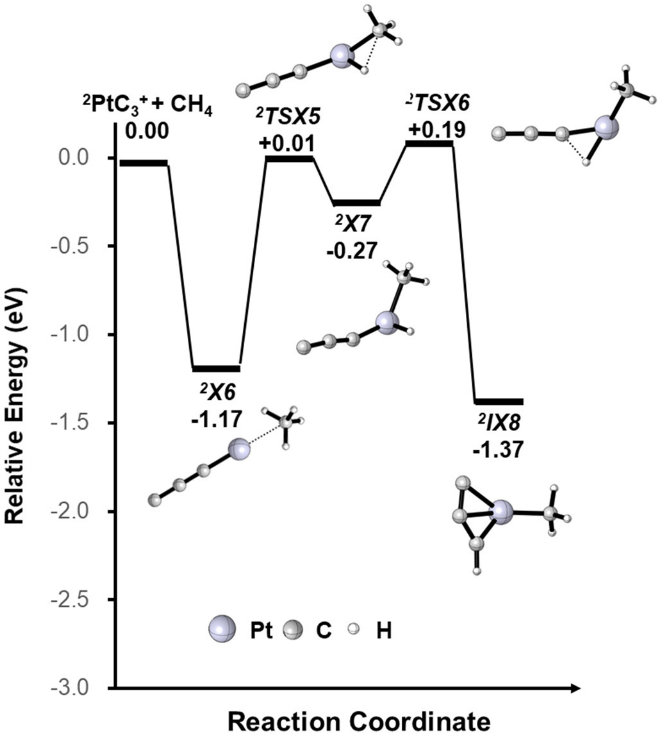

Figure S6. The other possible potential energy profiles of the reaction of PtC_3^+ and CH_4. The energies are given in eV.

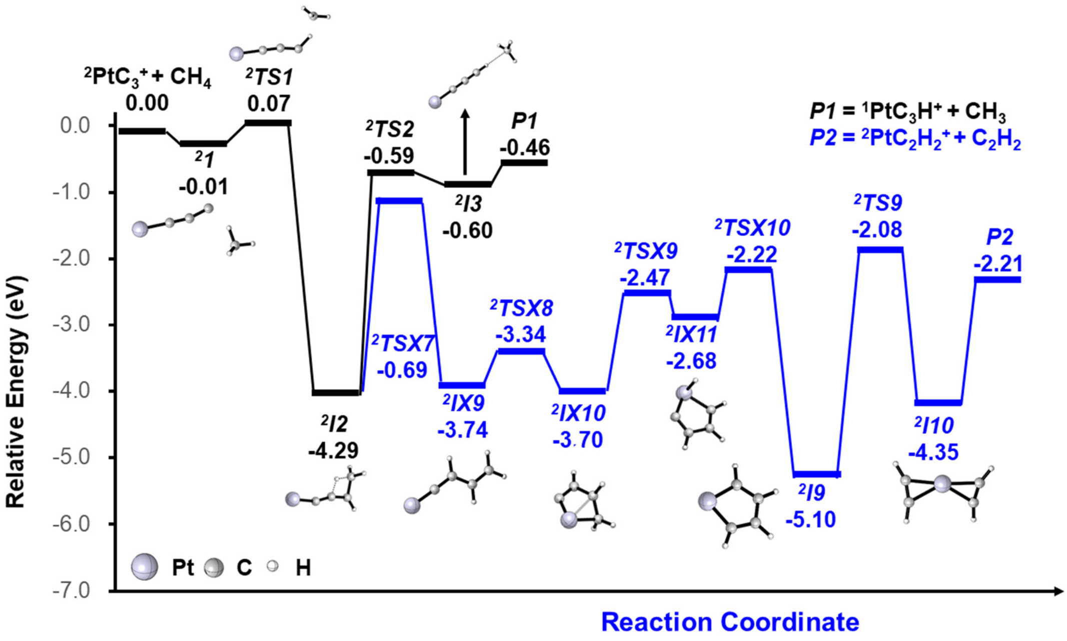

Figure S7. The other possible potential energy profiles of the reaction of PtC_3^+ and CH_4. The energies are given in eV.
